# Maternal immune activation downregulates schizophrenia genes in the foetal mouse brain

**DOI:** 10.1093/braincomms/fcab275

**Published:** 2021-11-15

**Authors:** Lahiru Handunnetthi, Defne Saatci, Joseph C Hamley, Julian C Knight

**Affiliations:** 1 Wellcome Centre for Human Genetics, University of Oxford, Oxford OX3 9DU, UK; 2 Nuffield Department of Clinical Neurosciences, John Radcliffe Hospital, University of Oxford, Oxford OX3 9DU, UK; 3 Nuffield Department of Primary Care Health Sciences, Radcliffe Observatory Quarter, University of Oxford, Oxford OX2 6GG, UK

**Keywords:** schizophrenia, genetics, maternal, immune, infection

## Abstract

Susceptibility to schizophrenia is mediated by genetic and environmental risk factors. Maternal immune activation by infections during pregnancy is hypothesized to be a key environmental risk factor. However, little is known about how maternal immune activation contributes to schizophrenia pathogenesis. In this study, we investigated if maternal immune activation influences the expression of genes associated with schizophrenia in foetal mouse brains. We found that two sets of schizophrenia genes were downregulated more than expected by chance in the foetal mouse brain following maternal immune activation, namely those genes associated with schizophrenia through genome-wide association study (fold change = 1.93, false discovery rate = 4 × 10^−4^) and downregulated genes in adult schizophrenia brains (fold change = 1.51, false discovery rate = 4 × 10^−10^). We found that these genes mapped to key biological processes, such as neuronal cell adhesion. We also identified cortical excitatory neurons and inhibitory interneurons as the most vulnerable cell types to the deleterious effects of this interaction. Subsequently, we used gene expression information from herpes simplex virus 1 infection of neuronal precursor cells as orthogonal evidence to support our findings and to demonstrate that schizophrenia-associated cell adhesion genes, *PCDHA2, PCDHA3* and *PCDHA5*, were downregulated following herpes simplex virus 1 infection. Collectively, our results provide novel evidence for a link between genetic and environmental risk factors in schizophrenia pathogenesis. These findings carry important implications for early preventative strategies in schizophrenia.

## Teaser

Handunnetthi et al. find that schizophrenia genes are downregulated by maternal immune activation mimicking viral infections. These downregulated genes play key roles in neurodevelopmental processes such as neuronal cell adhesion. Overall, these findings provide insight into how genetic and environmental factors may interact in the causal cascade to schizophrenia.

## Graphical Abstract

**Graphical Abstract:**
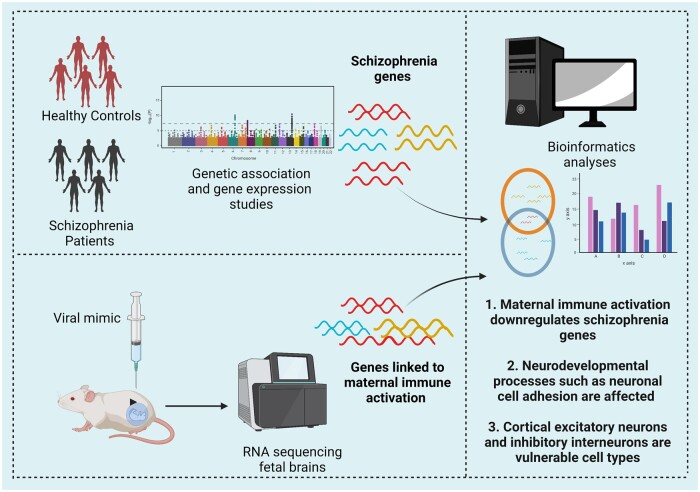

## Introduction

Epidemiological studies show overwhelming evidence that both genetic and environmental risk factors are important in the aetiology of schizophrenia.^1^ Recent advances in genomics have provided unprecedented insights into the genetic risk factors. Firstly, genome-wide association studies (GWAS) have identified over two hundred independent genetic risk variants in schizophrenia.^2^ Many of these genetic risk variants map to genes involved in synaptic organization and neurodevelopment, shedding light on the underlying disease mechanisms. Secondly, transcriptomic studies have identified differentially expressed genes in schizophrenia brains compared to healthy controls.^3^^,^^4^ These gene expression signatures are cell-type-specific and include genes that are important for neurodevelopmental processes.

Although the effect of the environment in schizophrenia is not mediated by a single factor, maternal infections during gestation are implicated.^5^ Epidemiological associations between maternal exposure to infections in gestation and subsequent increased risk of schizophrenia are established.^6–11^ Intriguingly, maternal infection in gestation and family history of psychiatric disorder show a synergistic increase in the risk of psychosis in the offspring.^12^^,^^13^ This points towards a possible gene–environment interaction that is yet to be discovered. Furthermore, the role of maternal infections in schizophrenia has been explored extensively using maternal immune activation (MIA) models.^14^ For example, offspring from mice exposed to polyinosinic:polycytidylic acid (polyI:C), a synthetic double-stranded RNA molecule that mimics viral infection, show pathological neurochemical and behavioural phenotypes that are characteristic of schizophrenia.^14^^,^^15^

However, our understanding of the mechanisms by which MIA contributes to increased risk of schizophrenia in the offspring in later life, and its role in gene–environment interactions remain poorly understood. Therefore, the aim of this study was to examine the relationship between recently discovered genetic risk factors in schizophrenia and the deleterious environmental effect of MIA in gestation using multiple genomic datasets. Specifically, this study investigated whether schizophrenia-associated genes were differentially expressed in foetal brains following MIA and then determined if any particular brain cell types and biological processes were affected by the interplay between MIA and schizophrenia-associated genes..

## Materials and methods

### Schizophrenia-associated genes from GWAS

Two sets of schizophrenia-associated genes were extracted from the largest GWAS to date comprising of 69 369 patients and 236 642 controls.^2^ The first set included genes (*n* = 130) that are likely to be disease causal identified through multiple fine-mapping approaches. The second set covered a broad group of credible genes (*n* = 644) with some genomic support for their role in the disease. We also extracted fined mapped genes identified through GWAS from the Open Targets Platform^16^ for bipolar disorder (BD), autism spectrum disorders (ASD) and Alzheimer’s disease representing comparative psychiatric, neurodevelopmental and neurodegenerative disorders, respectively.

### Differentially expressed genes in schizophrenia brains

Cell-type-specific gene expression patterns linked to schizophrenia were obtained from a single-cell RNA-sequencing study of 24 schizophrenia and 24 age- and sex-matched healthy brains.^3^ The tissue samples were derived from the prefrontal cortex of post-mortem brains with single cell libraries generated using the 10× Genomics Chromium Platform and sequenced using an Illumina NextSeq500 machine. Cell-type-specific gene expression patterns were available for 18 cell types, including excitatory cortical neurons, GABAergic interneurons, astrocytes, oligodendrocyte progenitor cell, microglia and endothelial cells in schizophrenia brains. Overall, there were 1637 upregulated and 2492 downregulated genes in schizophrenia brains compared to controls across all cell types.

### Maternal viral-like immune activation and gene expression in foetal brains

Induction of MIA by polyI:C (i.e. simulating viral infection) in gestation leads to behavioural and cognitive dysfunctions relevant to schizophrenia in the offspring.^15^ Gene expression changes in foetal brains following polyI:C administration were extracted from a published dataset.^15^ In this study, we used the data from the study by Tsivion-Visbord et al. for several reasons. First, transcriptomic profiling through RNA sequencing provides comprehensive information on gene expression changes in the foetal brain. Second, 24 h or more from immune insult to foetal brain transcriptomic profiling is important to investigate lasting effects on gene expression. Third, earlier exposure to immune activation in gestation is likely to be relevant for schizophrenia based on epidemiological evidence.

Briefly, we analysed data from pregnant mice injected with polyI:C (5 mg/kg/ml) (*N* = 8) or saline (*N* = 11) into the tail vein on gestational day 9 (time point analogous to the end of first trimester of human gestation, which has previously been implicated as a risk period for infection and subsequent development of schizophrenia^10^). After 24 h, the pregnant mice had been sacrificed and foetal brains extracted for transcriptomic analysis. Total RNA was extracted using the RNeasy Mini kit and sequenced using the NextSeq 500 Sequencing machine. Subsequently, differentially expressed genes (defined as >1.5 log_2_FC, FDR <0.05) were converted to human orthologues for downstream analyses using the BioMart database.^17^

### HSV-1 infection induced gene expression changes in neuronal precursor cells

Direct inflammatory insult to neuronal precursor cells by infection with Herpes Simplex Virus 1 (HSV-1) results in impaired neural differentiation and cortical layer organization in a cellular model of neurodevelopment.^18^ We extracted gene expression data from this model and published dataset to investigate the effects of HSV-1 infection on schizophrenia-associated genes. In this model, induced pluripotent stem cell-derived neuronal precursor cells were infected with HSV-1 MOI of 0 (control), 0.2 (low HSV) and 2 (high HSV). The cells were harvested after 72 h and RNA was extracted for transcriptomic analysis. RNA sequencing was performed using an Illumina NextSeq 6000 and differentially expressed genes were defined as >1.5 log_2_FC, FDR <0.05.

### Statistical analyses

We tested if schizophrenia genes were enriched among up or downregulated genes in the foetal brain following MIA using a hypergeometric test. The minimum overlap was set to five and the *P*-values were adjusted for multiple testing by controlling the false discovery rate (FDR). Next, the enriched genes were mapped to known Gene Ontology biological processes and cellular components to provide mechanistic insight.^19^ This was based on identifying biological processes and cellular components whose associate genes mapped to enriched genes (between schizophrenia and MIA genes) using the Fisher’s exact test. Also, we tested if the enriched genes mapped to any specific brain cell types using a previously described RNA-sequencing dataset of 24 brain cell types.^20^ Only genes in the top decile for cell specificity (defined as total expression of a gene in one cell type compared to that in all cell types) were considered in our analysis. All gene enrichment analyses were conducted using the xEnricher functions in the R package ‘XGR’ (version 1.1.4).^21^ Lastly, ANOVA with repeated measures was used to compare the expression of genes of interest between control, low HSV and high HSV groups while a *t*-test was used to compare between two of these groups.

### Data availability

MIA gene expression data sets used in this study are available from the NCBI Sequence Read Archive (accession code: PRJNA602886) and schizophrenia brain MULTI-seq data are available at Synapse (https://www.synapse.org Accessed 20 November 2021). Other data are available from the corresponding authors upon reasonable requests.

## Results

### Schizophrenia GWAS genes are downregulated in the foetal brain following MIA

**Figure 1:**
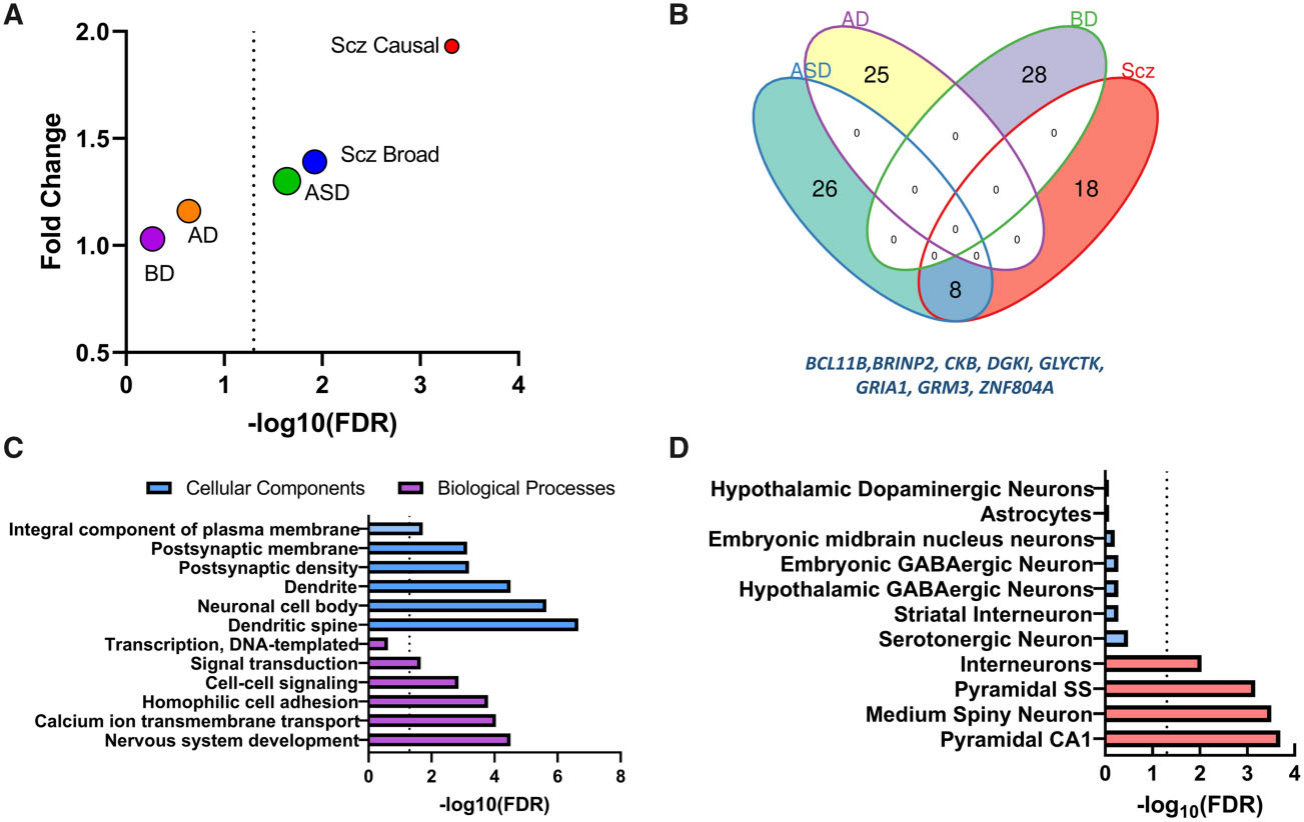
**Schizophrenia GWAS genes and maternal immune activation**. (**A**) Enrichment of GWAS-associated genes among differentially expressed foetal genes following MIA. ASD = autism spectrum disorder genes; AD = Alzheimer’s disease genes; BD = bipolar disease genes; Scz causal = schizophrenia genes identified through multiple fine mapping approaches; Scz Broad = credible schizophrenia genes with some genomic evidence. The size of the circles represents the number of overlapping genes. The dashed line is set at FDR = 0.05. (**B**) The intersect of MIA genes between ASD, Alzheimer’s disease, BD and Scz. The eight shared genes between ASD and schizophrenia are listed in blue. (**C**) Mapping of enriched schizophrenia genes to cellular components (blue) and biological processes (purple) in Gene Ontology. The dashed line is set at FDR = 0.05. (**D**) Mapping of enriched schizophrenia genes to brain cell types using RNA-sequencing dataset. The dashed line is set at FDR = 0.05.

We first investigated if GWAS-associated schizophrenia genes were up or downregulated more than expected by chance following MIA. We found a significant enrichment of schizophrenia GWAS genes among downregulated genes in the foetal brain following MIA, both for reported likely causal GWAS genes (*n* = 130) (gene set 1) (fold change = 1.93, FDR = 0.0004) and reported credible GWAS genes (*n* = 674) (gene set 2) (fold change = 1.39, FDR = 0.012) (Fig. 1A). There was no enrichment of schizophrenia genes among upregulated foetal genes following MIA. Additionally, we found a significant enrichment of ASD-associated genes (fold change = 1.3 FDR = 0.023) among MIA-induced downregulated genes in the foetal brain. Eight genes were shared between schizophrenia and ASD (Fig. 1B). By contrast, we found no significant enrichment for BD (fold change = 1.03 FDR = 0.54) or Alzheimer’s disease-associated genes (fold change = 1.16, FDR = 0.23). Also, we found no enrichment of Alzheimer’s disease, BD and ASD genes among the upregulated genes following MIA.

The enriched genes between schizophrenia and MIA mapped to several cellular components of neurons including dendritic spine (fold change = 27.5, FDR = 2.2 × 10^−7^), neuronal cell body (fold change = 11.9, FDR = 2.3 × 10^−6^) and postsynaptic density (fold change = 9.8, FDR = 6.5 × 10^−4^) (Fig. 1C). Furthermore, these genes mapped to biological processes (Fig. 1C), including nervous system development (fold change= 11, FDR =3.1 × 10^−5^), calcium ion transmembrane transport (fold change = 17.1, FDR = 9 × 10^−5^) and homophilic cell adhesion (fold change = 13.4, FDR = 1.6 × 10^−4^). We found that schizophrenia-associated members of the clustered protocadherin family *PCDHA2*, *PCDHA3* and *PCDHA5* were downregulated by MIA and mapped to the cell adhesion biological processes.

### Enriched schizophrenia GWAS genes map to specific brain cell types

We subsequently investigated if the MIA-schizophrenia GWAS (MIA-SG) gene set mapped to specific cell types using single cell RNA-seq data from 24 brain cell types. We found significant enrichments for neuronal cells, such as pyramidal CA1 neurons (fold change = 2.65, FDR = 2 × 10^−4^), pyramidal SS neurons (fold change = 2.2, FDR = 1.9 × 10^−4^) medium spiny neurons (fold change = 4.75, FDR = 3.1 × 10^−4^) and interneurons (fold change = 2.23, FDR = 0.0092) (Fig. 1D). Genes *GRIA1* and *GRM3* encoding glutamate receptor subunits mapped to the pyramidal cells. We did not find any enrichment for other brain cell types, such as serotonergic neurons, oligodendrocytes or microglia (Fig. 1D).

### Differentially expressed genes in schizophrenia brains are downregulated in the foetal brain following MIA

**Figure 2:**
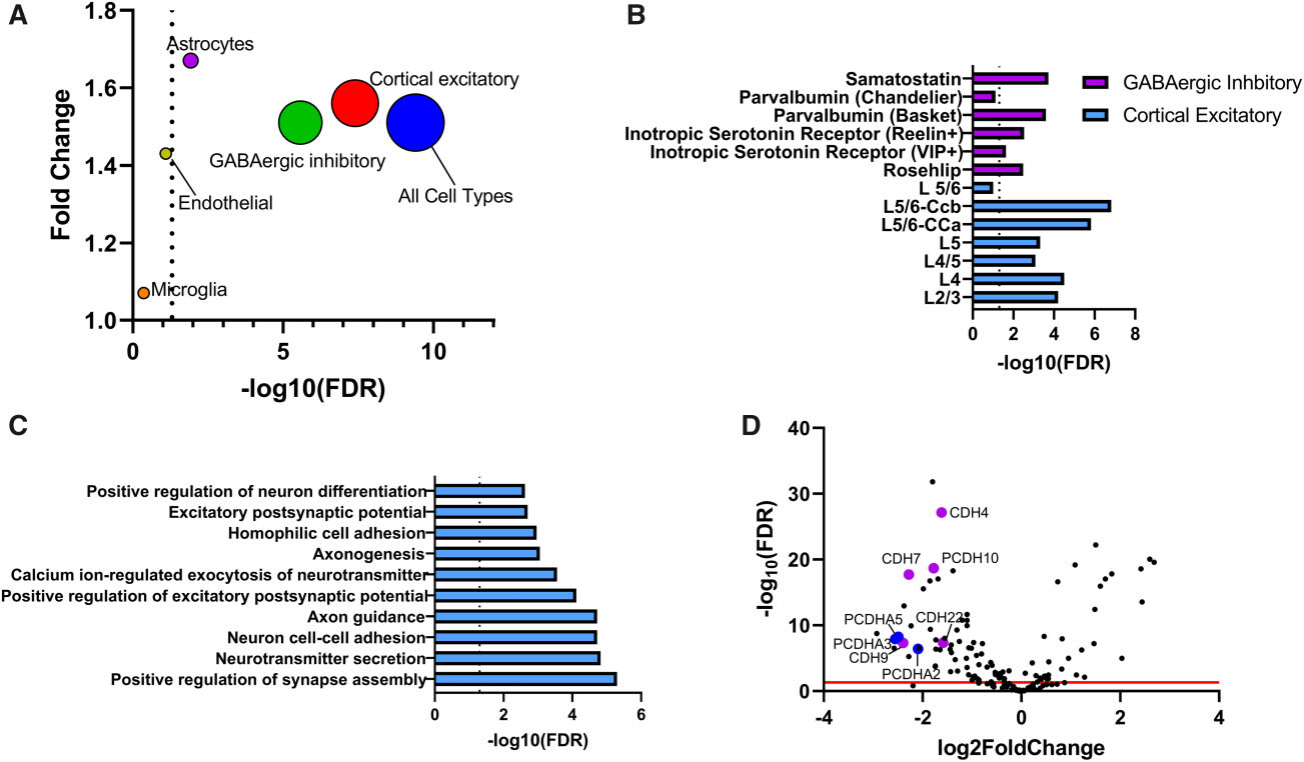
**Schizophrenia gene expression signature and maternal immune activation**. (**A**) Enrichment of downregulated genes in schizophrenia among downregulated genes in foetal brains following MIA. Single-cell RNA-sequencing data from schizophrenia were used to test enrichment in specific brain cell types (labelled). The size of the circles represents the number of overlapping genes. The dashed line is set at FDR = 0.05. (**B**) Breakdown of GABAergic inhibitory interneurons cell types (purple) and cortical excitatory cell types (blue). (**C**) Mapping of enriched schizophrenia genes to biological processes in Gene Ontology. The dashed line is set at FDR = 0.05. (**D**) Volcano plot showing expression levels of genes involved in cell adhesion (GO biological process.) following MIA. Genes labelled in blue are GWAS-associated schizophrenia genes whereas genes labelled in purple are downregulated genes in schizophrenia brains. The red line is set at FDR = 0.05.

We next explored the interaction between schizophrenia genes and MIA using an independent set of genes relevant to schizophrenia. Specifically, we investigated if differentially expressed genes in adult schizophrenia brains were enriched among up or downregulated foetal genes following MIA, which we refer to as the MIA-schizophrenia differentially expressed (MIA-SDE) gene set. Overall, there was a significant enrichment of downregulated schizophrenia genes among MIA-induced downregulated genes (fold change = 1.51, FDR = 4 × 10^−10^) (Fig. 2A). We did not find a significant enrichment of upregulated schizophrenia genes among upregulated MIA genes. The observed enrichment was specific to brain cell types including cortical excitatory (fold change = 1.56; FDR = 4.1 × 10^−8^), GABAergic interneurons (fold change = 1.51; FDR = 2.7 × 10^−6^) and astrocytes (fold change = 1.67; FDR = 0.012). Further subdivision of the cortical excitatory cell identified cortico-cortical projections in layer 5/6 (fold change = 1.68; FDR = 1.5 × 10^−7^) whereas subdivision of the GABAergic inhibitory cells identified somatostatin (fold change = 1.51; FDR = 2.7 × 10^−6^) and parvalbumin-expressing GABAergic interneurons (fold change = 1.71; FDR = 2.5 × 10^−5^) as the most significant cell types (Fig. 2B).

The enriched genes across all cell types mapped to several cellular components of neurons including excitatory synapse (fold change = 40.4, FDR = 2.6 × 10^−9^), postsynaptic density (fold change = 7.89, FDR = 1.9 × 10^−6^) and dendritic spine (fold change = 8.86, FDR = 2.9 × 10^−5^). Furthermore, these genes mapped to key biological processes in the brain including positive regulation of synapse assembly (fold change = 15.7, FDR = 5 × 10^−6^), axon guidance (fold change = 7.42, FDR = 1.9 × 10^−5^), positive regulation of excitatory postsynaptic potential (fold change = 21.2, FDR = 7.7 × 10^−5^), calcium ion regulated exocytosis of neurotransmitter (fold change = 13.5, FDR = 2.8 × 10^−4^) and homophilic cell adhesion (fold change = 5.2, FDR = 0.0011). Schizophrenia genes relevant to cell adhesion comprised of several members of the cadherin family *CDH22*, *CDH4*, *CDH7* and *CDH9* as well as the protocadherin *PCDH10* (Fig. 2D).

### HSV infection alters the expression of schizophrenia genes involved in cell adhesion during neurodevelopment

**Figure 3:**
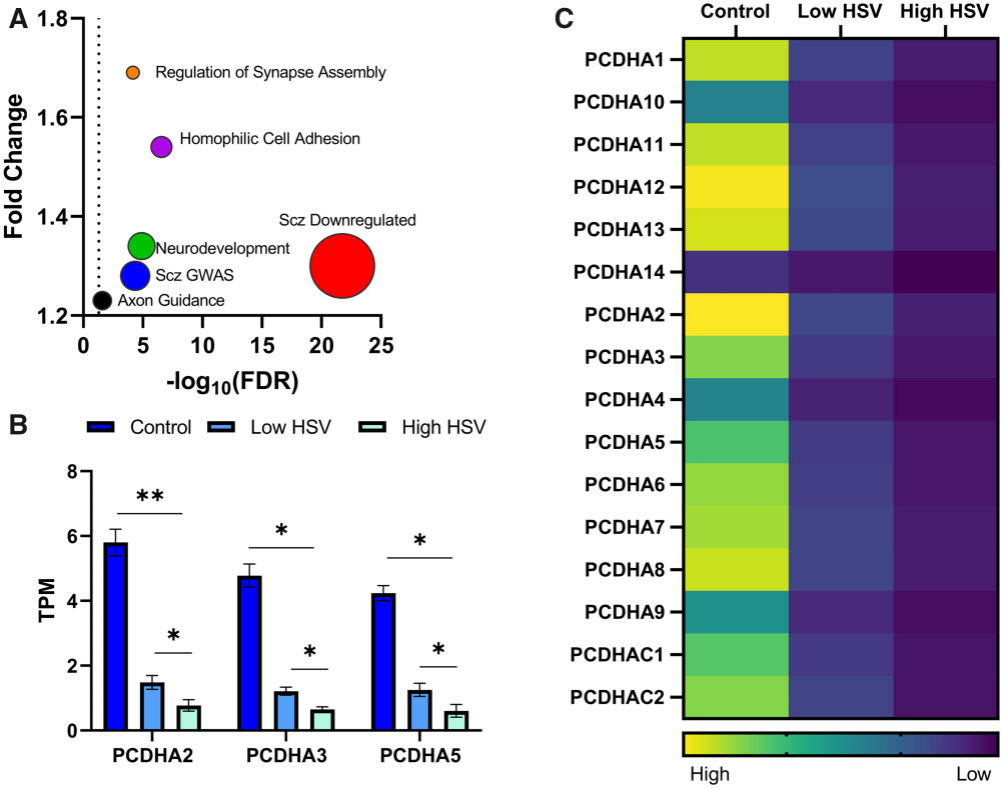
Schizophrenia genes and HSV-1 infection. (**A**) Enrichment of schizophrenia genes and members of relevant GO biological processes among downregulated genes in neuronal precursor cells following HSV-1 infection. The size of the circles represents the number of overlapping genes. The dashed line is set at FDR = 0.05. (**B**) Bar plots showing the effect of HSV-1 infection on the expression of schizophrenia associated *PCDHA2, PCDHA3* and *PCDHA5.* TPM = transcripts per million. **P < 0.001 and *P < 0.05. ANOVA with repeated measures was used to compare the expression of genes of interests between control, low HSV and high HSV groups while a *t*-test was used to compare between two of these groups. (**C**) Heat map showing the gene expression of clustered protocadherin alpha family members following high HSV-1 and low HSV-1 infection in neuronal precursor cells.

Next, we investigated the effect of HSV-1 infection in neuronal precursor cell on the expression of schizophrenia-associated genes. We found that both MIA-SG genes (fold change = 1.28, FDR = 4.8 × 10^−5^) and MIA-SDE genes (fold change = 1.3, FDR = 1.8 × 10^−22^) were significantly downregulated in neuronal precursor cells following HSV-1 infection (Fig. 3A). In addition, we tested if gene members of biological processes identified in earlier analyses were enriched among differentially expressed genes following HSV-1 infection. We found significant enrichment for nervous system development (fold change = 1.34, FDR = 1.3 × 10^−5^), homophilic cell adhesion (fold change = 1.54, FDR = 2.8 × 10^−7^), axonal guidance (fold change = 1.23, FDR = 0.026) and regulation of synaptic assembly (fold change = 1.69, FDR = 7 × 10^−5^) among downregulated genes (Fig. 3A). Subsequently, we tested the effects of HSV-1 infection on the expression of schizophrenia-associated protocadherins *PCDHA2, PCDHA3* and *PCDHA5* given their identification in cell adhesion biological processes in our earlier analysis. We found that all three genes were significantly downregulated following HSV-1 infection in a dose-dependent manner; *PCDHA2* (control versus low: *P* = 0.001; low versus high: *P* = 0.012), *PCDHA3* (control versus low: *P* = 0.002; low versus high: *P* = 0.003) and *PCDHA5* (control versus low: *P* = 0.002; low versus high: *P* = 0.02) (Fig. 3B).

## Discussion

This study demonstrates that maternal immune activation results in downregulation of schizophrenia-associated genes in the foetal brain, highlighting an important link between genetic and environmental risk factors in the disease. We used two independent sets of schizophrenia genes and MIA-induced expression changes in foetal mouse brains to discover this biological interplay between genetic risk factors and immune activation in schizophrenia. Subsequently, gene expression data from HSV infection of neuronal precursor cells were used to provide independent evidence in support of our findings. Collectively, our results support the hypothesis that MIA can affect genetically determined foetal brain development that later increase the risk of schizophrenia. We can extrapolate that the combination of mutations in neurodevelopment genes and MIA can synergistically increase the risk of schizophrenia. This is consistent with the previously observed statistical interaction between maternal infection in gestation and family history of psychiatric disorders in determining the risk of psychosis.^12^^,^^13^ Furthermore, our findings are supported by previous studies showing that immune activation of stem cell-derived neurons from healthy individuals result in downregulation of schizophrenia genes^22^ and co-culture of stem cell-derived neurons with immune activated microglia give rise to lasting changes in schizophrenia patient interneurons.^23^

We found that genes associated with ASD were also downregulated in the foetal brain following MIA; however, this was not the case for BD and Alzheimer’s disease. Our findings are consistent with observations that maternal infection is a risk factor for ASD^24^ and that MIA affects the expression of ASD genes relevant to neurodevelopment.^25^ Therefore, it is possible that the interaction between genetic risk factors and MIA represents a common disease mechanism for neurodevelopmental disorders. Indeed, *ZNF804*, a shared gene between schizophrenia and ASD, has been previously shown to be important in neuronal progenitor proliferation and migration^26^ as well as in synaptic organization and dendritic spine density.^27^ Furthermore, *GRIA1* and *GRM3* encoding subunits of the glutamate receptors were shared between schizophrenia and ASD, suggesting that MIA could affect glutamatergic transmission in both of these disorders.

Our results demonstrate that the deleterious effects of this interaction between schizophrenia genes and MIA are likely to be cell-type-specific. We found that schizophrenia genes downregulated by MIA mapped to cortical excitatory neurons and inhibitory interneurons. This was consistent between both GWAS and differentially expressed genes in schizophrenia, providing strong support for susceptibility of these cell types to the deleterious effects of this interaction. Intriguingly, subdivision of GABAergic interneurons identified somatostatin and parvalbumin-expressing GABAergic interneurons, whereas subdivision of cortical excitatory neurons identified cortico-cortical projections in layer 5/6 as the most vulnerable cell types. These findings complement previous observations that MIA by polyI:C leads to aberrant excitatory synaptic transmission^28^ as well as delays to excitatory-to-inhibitory GABA switch during neurodevelopment.^29^ Crucially, knowledge of cell types that are susceptible to the deleterious effects of this interaction between schizophrenia genes and MIA will help to guide future *in vitro* studies. Although we highlight neuronal cell types in which schizophrenia genes are likely to be affected by MIA leading to functional effects, this does not exclude the relevance of other brain cell types in the causal cascade to schizophrenia. This is notion further supported by our observation that genes downregulated by MIA mapped to astrocytes highlighting the relevance of this cell type to in MIA and schizophrenia. Further work is need to understand how MIA can affect these cell types such as microglia and astrocytes.

Furthermore, our results demonstrate that schizophrenia genes downregulated by MIA map to specific biological processes and cellular components providing insight into how this interaction could contribute to the causal cascade. First, the enriched genes mapped to cellular components such as the dendritic spine and post synaptic density, as well as biological processes involving synaptic assembly. In line with our findings, previous studies in MIA models have shown reduced cortical dendritic spine density, turnover, and connectivity, as well as altered synaptic transmission.^30^ Second, the enriched schizophrenia genes mapped to neurodevelopmental processes, such as axonal guidance and neuronal cell adhesion. This complements previous findings that both MIA in mice and HSV infection of human neuronal precursor cells result in dysregulated neurogenesis.^18^^,^^31^ Third, we found that several members of the protocadherin and cadherin families known to be important for cell adhesion processes were downregulated by MIA. Additionally, we showed that schizophrenia-associated protocadherins *PCDHA2, PCDHA3* and *PCDHA5* were downregulated in a dose-dependent manner in neuronal precursor cells following HSV infection. These findings are consistent with previous observations that stem cell-derived neurons from schizophrenia patients had dysregulated expression of protocadherin genes and mice lacking protocadherin-α showed defective structural and synaptic density in the prefrontal cortex.^32^

This study had several limitations including translational differences between mice and humans and the inability of polyI:C to fully recapitulate the complexity of immune responses in humans during pregnancy. Furthermore, our analyses were restricted by the quality of available genomic datasets. For example, Tsivion-Visbord et al. used whole brains to study gene expression patterns following polyI:C administration, which in turn could have led to loss of cell-type-specific gene expression information. Future studies examining gene expression patterns in single cells will shed more light on this topic. In this study, we could not comprehensively examine the role of sex given the small numbers each group. Therefore, further studies investing how sex contributes to MIA induced genes expression changes and its relationship to the pathogenesis of schizophrenia are warranted. Another limitation was that we only investigated MIA-induced gene expression changes in one model of MIA. Therefore, further work is needed to understand how other models of MIA involving various immune agents and gestational time points could affect the expression of schizophrenia genes.

This study provides novel insight into the interplay between genetic and environmental factors in schizophrenia and highlights gestation as a critical window of vulnerability. Several lines of genomic evidence converge on the observation that MIA leads to dysregulation of schizophrenia genes that are important in neurodevelopmental and synaptic assembly processes. Further studies are needed to understand exactly how this interaction could lead to long-term alterations of the offspring brain structure and how the notions of ‘risk and resilience’ to MIA will affect schizophrenia genes.^33^ In addition, studies investigating the role of protocadherins at protein level are needed to understand their role is the schizophrenia pathogenesis. Importantly, these findings carry clear implications for disease prevention strategies in schizophrenia because treatment and/or prevention of maternal infection during pregnancy is a tractable health goal.

## Abbreviations

ASD =: autism spectrum disorders
BD =: bipolar disorder
FDR =: false discovery rate
GWAS =: genome-wide association studies
HSV =: Herpes Simplex Virus
MIA =: maternal immune activation
MIA-SDE =: MIA-schizophrenia differentially expressed
MIA-SG =: MIA-schizophrenia GWAS
polyI:C =: polyinosinic:polycytidylic acid
